# CASHeart: A database of single cells chromatin accessibility for the human heart

**DOI:** 10.1002/qub2.90

**Published:** 2025-02-12

**Authors:** Qun Jiang, Xiaoyang Chen, Zijing Gao, Jinmeng Jia, Shengquan Chen, Rui Jiang

**Affiliations:** ^1^ Ministry of Education Key Laboratory of Bioinformatics Bioinformatics Division at the Beijing National Research Center for Information Science and Technology Center for Synthetic and Systems Biology Department of Automation Tsinghua University Beijing China; ^2^ School of Mathematical Sciences and LPMC Nankai University Tianjin China

**Keywords:** human heart, chromatin accessibility, single cell, database

## Abstract

Human heart single‐cell chromatin accessibility data reveal the diversity and complexity of heart cells at the epigenomic level, providing a detailed perspective for understanding the molecular mechanisms of heart development, function maintenance, disease occurrence, and therapeutic response. However, the current human heart single‐cell chromatin accessibility data are relatively scarce, lacking large‐scale, high‐quality, and integrated datasets. To facilitate research and utilization, we have established a comprehensive database of human heart single‐cell chromatin accessibility data, CASHeart. This database collects sequencing fragment files from publicly available papers, processes and counts data for 212,600 human heart cells, and provides transformed gene activity scores. All data are accessible for browsing and download via the online platform. We demonstrate that the data provided by CASHeart reveal heart cell type heterogeneity more effectively than the original data, aiding in the analysis of differentially accessible chromatin regions and activated genes. Moreover, we show that the incorporation of single‐cell chromatin accessibility data and transformed gene activity scores from CASHeart as reference datasets enhances the analysis of heart single‐cell epigenomic and transcriptomic data, whereas the unified chromatin accessible regions provided by CASHeart can assist in the study of gene regulation and genetic variation in human cardiac cells.

## 1 INTRODUCTION

The human heart is a vital organ essential for sustaining life, with its complex biological functions and significant medical importance making it a central focus of biomedical research [1]. Understanding the development, function, and pathological changes of the heart has profound implications for advancing the diagnosis, treatment, and prevention of cardiovascular diseases [2]. In this context, the study regarding chromatin accessibility data in the heart is particularly crucial, as such data are not only essential for revealing epigenetic characteristics of the cardiac genome but also indispensable for deciphering cell type‐specific regulatory networks, understanding the molecular basis of cardiac diseases, and identifying new therapeutic targets [3, 4, 5].

With the advancement of single‐cell sequencing technologies, several studies have been published on human heart single‐cell chromatin accessibility [6]. For instance, Kai and Silvia et al. used sci‐ATAC‐seq technology to map chromatin accessibility across major tissues in adult and pediatric humans, including the human heart tissue [7, 8]. Kanemaru et al. analyzed the spatially resolved multi‐omics of the human cardiac niche, which includes chromatin accessibility landscapes of major cardiac cell types [9]. Ameen et al. generated single‐cell chromatin accessibility maps of fetal human heart tissue to define the multi‐cellular epigenomic and transcriptomic landscape of human heart cell development [10]. Although these studies provide valuable single‐cell chromatin accessibility data for epigenetic research on the human heart, some data are mixed with other tissues or omics datasets, and issues such as genomic inconsistency and batch effects pose challenges. Therefore, an extensive and uniformly defined database for integrated analysis is needed.

To facilitate the study of epigenetics and gene regulation in human heart tissues, this paper introduces CASHeart, a comprehensive database for human heart single‐cell chromatin accessibility. CASHeart collects sequencing fragment files from publicly available studies, aligns them to a unified genome version, performs consistent peak calling, and annotates cell types and sample identifiers based on the original studies’ annotation files. The database also includes batch correction and other processing steps, resulting in a standardized and integrated chromatin accessibility database. To enhance integrative analysis with epigenomic and transcriptomic data, we have also converted the processed single‐cell chromatin accessibility data into single‐cell gene activity scores using genomic annotation. An online platform has been developed to improve data accessibility and usability, allowing users to browse and download the data. CASHeart currently includes data from 56 samples, encompassing a total of 212,600 cells, and provides cell type‐specific chromatin accessibility differential analysis and differential gene expression analysis. We demonstrate that the uniformly processed single‐cell chromatin accessibility data outperforms the original data in revealing the specificity of chromatin accessibility in cardiac cells. To provide a perspective at the level of gene expression, we transformed the single‐cell chromatin accessibility data from CASHeart into gene activity scores. By introducing single‐cell chromatin accessibility data and converted gene activity scores as reference datasets, we improved the dimensionality reduction and clustering results of external datasets, confirming the effectiveness of the database. Finally, we also demonstrated that CASHeart can facilitate the analysis of cardiac‐specific gene regulation and genetic variation.

## 2 RESULTS

### 2.1 Overview of CASHeart

**FIGURE 1:**
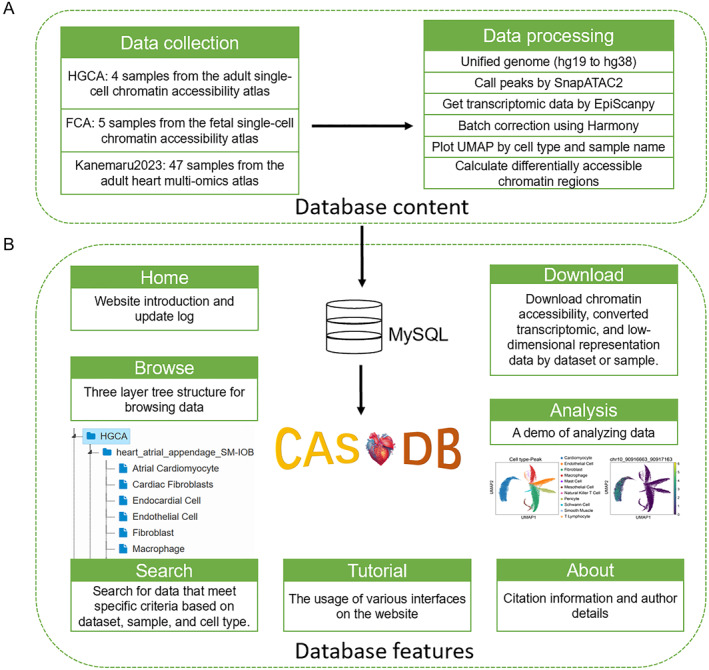
The framework of CASHeart. (A) Overview of the data collection and data processing workflow in the CASHeart Database. (B) Introduction to the main interfaces of the CASHeart website.

CASHeart consists primarily of two components: dataset construction and website development. As illustrated in Figure 1A, to construct the CASHeart dataset, we collected nine heart tissue samples from the single‐cell chromatin accessibility atlases constructed by Kai and Silvia et al. for adult and pediatric humans, respectively. The data from adults are referred to as HGCA, and those from infants as FCA, containing four and five samples of sequencing fragments files, respectively. In addition, we extracted chromatin accessibility data from the spatially resolved multi‐omics dataset of the human cardiac niche constructed by Kanemaru et al., obtaining fragments regarding a total of 47 samples. To ensure consistency in the genomic version, we converted genomic coordinates of all samples to GRCh38 (hg38). Next, we used the SnapATAC2 [11] toolkit to perform peak calling on the fragments files, where the built‐in MACS3 [12] method was used to assign uniform peak regions for all cells. We then utilized the gene activity score method in EpiScanpy along with the genomic annotation file to obtain the converted gene activity scores [13].

As shown in Figure 1B, the CASHeart online platform includes several main interfaces: Home, Browse, Search, Download, Tutorials, Analysis, and About. The Home interface provides a brief overview of the purpose of this platform, the data currently included, and the update log of the website. The Browse interface features a three‐tiered structure: datasets, samples, and cell types. When a dataset is selected, the right side displays UMAP [14] visualizations colored by cell type and sample for that dataset, along with matrix plots showing chromatin differential accessibility analysis across different cell types using EpiScanpy. When a sample is selected, UMAP visualizations colored by a cell type within the sample and matrix plots of chromatin differential accessibility analysis for different cell types in that sample are shown. When a cell type is selected, the top 25 cell type‐specific differentially accessible regions and specific expression genes are listed in a tabular format on the right. In the Search interface, users can select different datasets, samples, and cell types, and upon submitting a search query, the database returns detailed annotations for the data that meet the specified criteria in a tabular format. In the Download interface, users can download data with varying feature numbers by dataset or sample, as well as the converted gene activity scores. The Tutorials interface offers detailed instructions on how to use each interface within CASHeart, while the Analysis interface provides an example of how to perform analysis related to this database using Python. The About interface contains relevant information about the authors of the website.

### 2.2 CASHeart accurately reveals the heterogeneity of heart cell types

Different cell types within the heart exhibit cell type‐specific epigenetic characteristics, specifically in the regions of chromatin accessibility, which in turn lead to distinct data distributions after dimensionality reduction and clustering. To investigate the effectiveness of CASHeart data in revealing cell type specificity, we compared its clustering performance with that of the data provided in the original publications. For both the cell‐peak matrices from CASHeart and the original studies, cell types with fewer than 10 cells were filtered out. We then applied TF‐IDF [15] processing, performed dimensionality reduction using the default PCA [16] parameters in EpiScanpy, conducted batch correction across different samples using Harmony [17], and finally generated UMAP plots colored by cell type.

**FIGURE 2:**
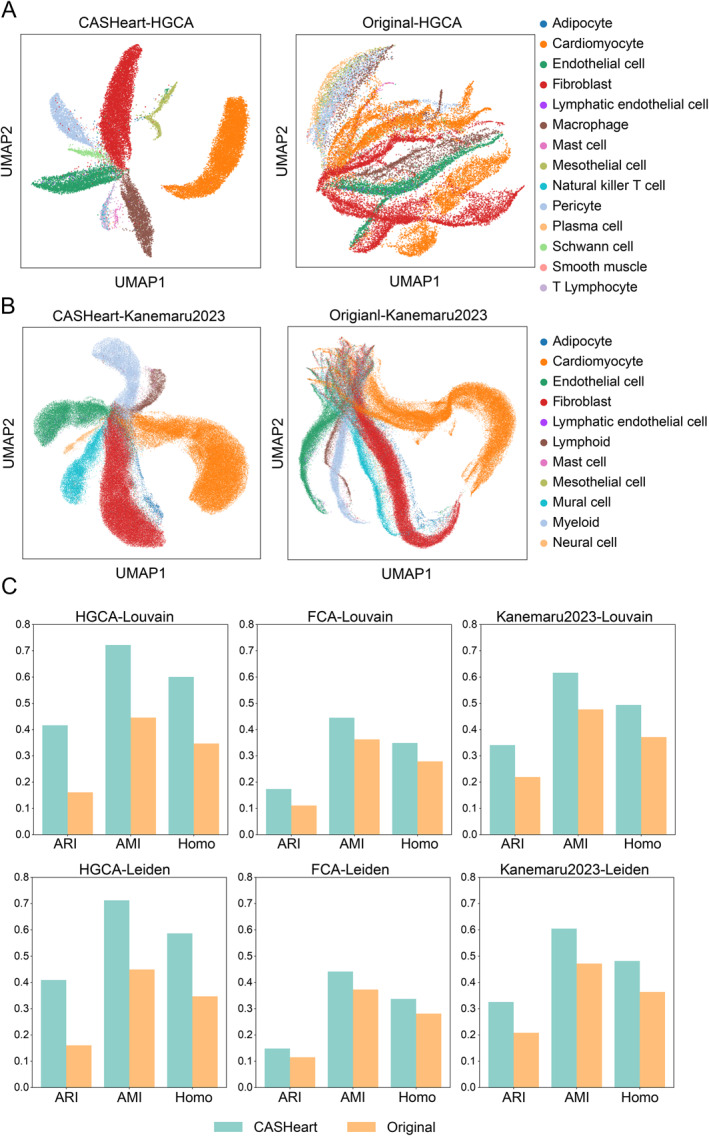
CASHeart better reveals the heterogeneity of cell types. (A) UMAP visualization comparison of the HGCA dataset in CASHeart and the original data with cell types having fewer than 10 cells filtered out. (B) UMAP visualization comparison of the Kanemaru2023 dataset in CASHeart and the original data with cell types having fewer than 10 cells filtered out. (C) Clustering metrics between Louvain clustering labels and true cell type labels, and between Leiden clustering labels and true cell type labels across the three datasets.

As shown in Figure 2A, the HGCA data provided by CASHeart effectively distinguishes between various cell types. It is worth noting that cardiomyocytes, which initially exhibited significant batch effects across four samples (Figure S1A), clustered together well after correction. In contrast, while the original HGCA dataset was able to reasonably distinguish cardiomyocytes, there was considerable overlap among non‐cardiomyocyte cells in the UMAP visualization before batch correction (Figure S1B). For more information on batch correction of CASHeart, see Figures S1C‐F. After batch correction performed by the sample, it led to a messy clustering effect, demonstrating instability. As depicted in Figure 2B, although the original Kanemaru2023 data already provided good separation of different cell types, CASHeart further improved the clustering, resulting in tighter grouping of cells of the same type. For example, CASHeart better distinguished the myeloid and lymphoid cell types, which were close to each other in the UMAP plot.

To quantitatively compare the clustering performance of the two datasets, we employed three common clustering metrics. The adjusted rand index (ARI) measures the similarity between two clustering results, taking into account the possibility of random matches and adjusting accordingly. The adjusted mutual information (AMI) is an information‐theoretic measure that evaluates the degree of information shared between two clustering results. Homogeneity (Homo) assesses the purity within clusters, that is, whether each cluster contains only true labels from a single category. To obtain these metrics, we used the Louvain and Leiden methods to derive clustering labels from the batch‐corrected data, and then compared these labels with the true labels to calculate the three clustering metrics [18]. As shown in Figure 2C, the three datasets from CASHeart outperform the corresponding original datasets in terms of clustering metrics using both Louvain and Leiden clustering methods.

### 2.3 CASHeart promotes the analysis of differentially accessible chromatin regions and activated genes

To study heart development, disease, and their underlying molecular mechanisms, a comprehensive understanding of gene expression patterns and chromatin states in cardiac cells is essential. However, the heterogeneity of cell types in such a complex organ as the heart adds difficulty to the analysis. Therefore, the precise characterization of differentially accessible chromatin regions and activated genes is crucial for unveiling the molecular basis of heart diseases and developing targeted therapeutic strategies [19]. Leveraging the extensive single‐cell chromatin accessibility data and converted gene activity scores within the database, the CASHeart online platform offers multi‐level analyses of differentially accessible chromatin regions and activated genes. On the CASHeart browsing interface, when users select a dataset or sample, the right panel provides results of such analysis for the corresponding data.

**FIGURE 3:**
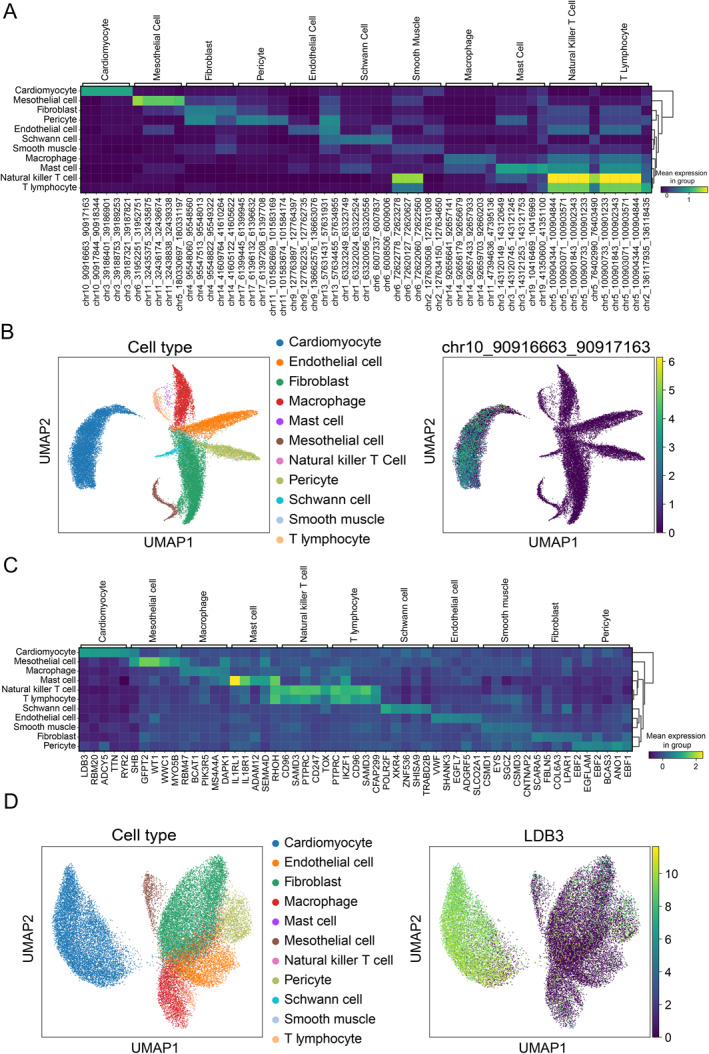
CASHeart promotes the analysis of differentially accessible chromatin regions and activated genes. (A) The accessibility status of the top 5 differentially accessible regions (ranked by *p*‐value) for each cell type in the HGCA dataset across all cell types. (B) UMAP visualization of chromatin accessibility data in the HGCA dataset: the left plot is colored by cell type, and the right plot is colored by the accessibility levels of the chromatin region chr10_90916663_90917163 across different cell types. (C) The activity levels of the top 5 differentially activated genes (ranked by *p*‐values) for each cell type in the HGCA dataset across all cell types. (D) UMAP visualization of the converted gene activity scores from the HGCA dataset: the left plot is colored by cell type, and the right plot is colored by the activity levels of the LDB3 gene across different cell types.

Figure 3A lists the average accessibility levels across all cell types for the top five differentially accessible regions among 11 cell types in the HGCA dataset. It is evident that the five differentially accessible regions in cardiomyocytes are nearly inaccessible in other cell types. The presence of these cell‐specific accessible regions leads to distinct separation of cardiomyocyte clusters from other cell types in the UMAP visualization. To further explore the genetic information underlying these cell‐specific differential regions, we searched the top cardiomyocyte‐specific accessible region, chr10_90916663_90917163, in the Ensembl Genome Browser [20] and found that it partially overlaps with an exon of the ANKRD1 gene. The ANKRD1 gene encodes a protein that regulates cardiac function and is associated with cardiomyopathy and heart failure [21]. The right panel of Figure 3B further illustrates the distribution of the accessibility of this region in cardiomyocytes. Additionally, it is notable that the consistent differentially accessible regions between closely related T lymphocytes and natural killer T cells lead to their early clustering in hierarchical clustering.

Figure 3C presents the top five differentially activated genes in each cell type from the gene activation perspective. For example, in cardiomyocytes, the top gene is LDB3, which encodes a protein known as ZASP that provides structural support and participates in the formation of cardiomyocyte junctional complexes [22]. The left panel of Figure 3D displays a UMAP plot colored by cell type, generated using converted single‐cell gene activity scores. Although not derived from actual transcriptomic data, this plot still distinguishes major cell types. The right panel of Figure 3D further demonstrates the specific expression of LDB3 in cardiomyocytes.

### 2.4 CASHeart can serve as a reference dataset to enhance the analysis of cardiac epigenomic and transcriptomic data

**FIGURE 4:**
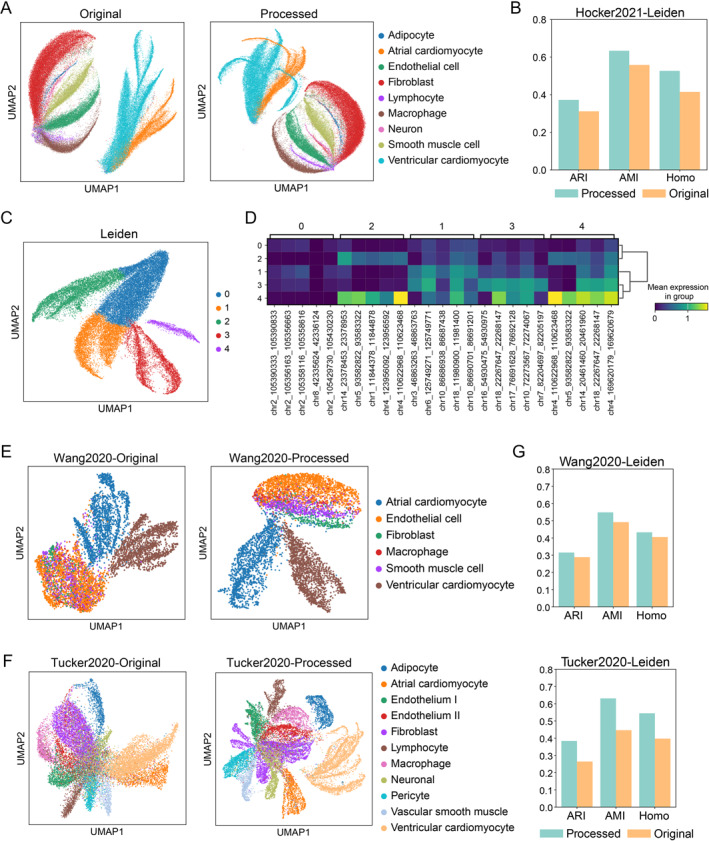
CASHeart can serve as a reference dataset to enhance the analysis of cardiac epigenomic and transcriptomic data. (A) Comparison of UMAP visualizations of the Hocker2021 dataset colored by cell type before and after incorporating CASHeart as a reference dataset. (B) Comparison of Leiden clustering metrics for the Hocker2021 dataset before and after incorporating CASHeart as a reference dataset. (C) UMAP visualization of cardiomyocytes in the Hocker2021 dataset, colored by Leiden clustering labels after incorporating the reference dataset. (D) The differential chromatin accessibility analysis of cardiomyocytes using *t*‐tests identified the top 5 peaks with the lowest *p*‐values for each Leiden cluster. (E) UMAP visualization of the Wang2020 dataset colored by cell type: the left panel shows the original data, while the right panel shows the results after incorporating gene activity scores from CASHeart as a reference dataset. (F) UMAP visualization of the Tucker2020 dataset colored by cell type: the left panel shows the original data, while the right panel shows the results after processing with gene activity scores from CASHeart as a reference dataset. (G) Comparison of clustering metrics before and after introducing the reference dataset for the Wang2020 and Tucker2020 datasets.

The incorporation of external reference datasets into the study of single‐cell ATAC‐seq (scATAC‐seq) data can substantially benefit such analysis as clustering and classification, where data are often limited in quantity and high in noise [23]. Reference datasets encompass a broader spectrum of cellular heterogeneity, offering a richer background for analysis and improving the identification of differentially accessible chromatin regions among various cell types [24]. To validate the effectiveness of CASHeart as a reference dataset for improving clustering performance in target datasets, we collected a new human heart single‐cell chromatin accessibility dataset, Hocker2021 [25]. Since we did not have access to the original sequencing files of this dataset, it was not included in CASHeart. We first mapped the peaks of Hocker2021 to those in the CASHeart database, and then performed peak selection, dimensionality reduction, and cluster analysis on the combined data (as detailed in Section 4.4). Figure 4A shows UMAP plots colored by cell type for the raw data and after introducing the reference dataset. Figure 4B compares three clustering metrics under these two scenarios. As observed, the inclusion of the reference dataset improved the separation of less abundant cell types in the Hocker2021 dataset, such as lymphocytes and macrophages, as well as adipocytes and neurons, which were otherwise difficult to distinguish in the original data.

In addition, atrial and ventricular cardiomyocytes exhibited distinct subtype distributions upon the introduction reference dataset. We further analyzed these cardiomyocytes, as shown in Figure 4C, where cardiomyocytes from the Hocker2021 dataset were jointly analyzed with those from CASHeart. Atrial and ventricular cardiomyocytes displayed clear subtype distributions. We applied the Leiden method for clustering and performed *t*‐tests to identify differential open chromatin regions across clusters. It is worth noting that clusters 2 and 4, both representing atrial cardiomyocytes, had similar differential open regions, but cluster 4 showed higher accessibility levels in most regions compared to cluster 2 (Figure 4D). A similar pattern was observed in ventricular cardiomyocytes, where cluster 0 had lower accessibility, while clusters 1 and 3 showed relatively higher accessibility. These findings indicate that incorporating reference datasets enables a more refined analysis of differential open chromatin regions within subtypes of the same cell type.

In single‐cell transcriptomics, data quality is crucial for the accuracy of analysis results. Low‐quality target datasets, often characterized by a small number of cells, high noise, or insufficient coverage, present significant challenges for data analysis [26]. Nevertheless, the incorporation of high‐quality external reference transcriptomic data can significantly improve these analysis outcomes [27, 28]. To validate the effectiveness of incorporating gene activity scores derived from CASHeart as a reference dataset, we collected two real human heart transcriptomic datasets for verification. First, the Wang2020 dataset explored the transcriptional differences between cardiomyocytes and noncardiomyocytes. In the original experiment, all non‐cardiomyocytes clustered together, and the distance between atrial and ventricular cardiomyocytes was relatively close (Figure 4E, left panel). After incorporating gene activity scores from CASHeart as a reference dataset, ventricular and atrial cardiomyocytes were well‐separated, and the previously clustered non‐cardiomyocytes displayed distinct stratification (Figure 4E, right panel). Second, Tucker2020 was a transcriptomic dataset comprising over 280,000 human heart cells, from which a subset of 20,000 cells was obtained through random sampling based on cell type. In the original data, atrial and ventricular cardiomyocytes could not be completely separated, and different subtypes of endothelial cells and macrophages were difficult to distinguish. However, after introducing CASHeart as a reference dataset, these issues were resolved, and ventricular cardiomyocytes and fibroblasts exhibited distinct subtypes (Figure 4F). To quantify the improvement in clustering performance from incorporating CASHeart gene activity scores, Figure 4G presents a comparison of clustering metrics before and after the introduction of CASHeart in the two datasets mentioned above. It is evident that after the incorporation of the CASHeart dataset, both datasets showed significant improvements across three clustering metrics. In conclusion, the introduction of gene activity scores from CASHeart as a reference dataset enhances clustering performance and facilitates the discovery of new subtypes in real single‐cell transcriptomic data from the heart.

### 2.5 CASHeart facilitates the analysis of cardiac‐specific gene regulation and genetic variation

To demonstrate how CASHeart can aid in elucidating gene regulation in cardiac cells, we performed analyses using Cicero [29], an R package for analyzing single‐cell chromatin accessibility experiments primarily by predicting *cis*‐regulatory interactions in the genome through the detection of shared accessibility. We calculated co‐accessibility of chromatin open regions in the single‐cell chromatin accessibility data from CASHeart, stratified by different cell types, to investigate the gene regulatory mechanisms specific to cardiac cells.

**FIGURE 5:**
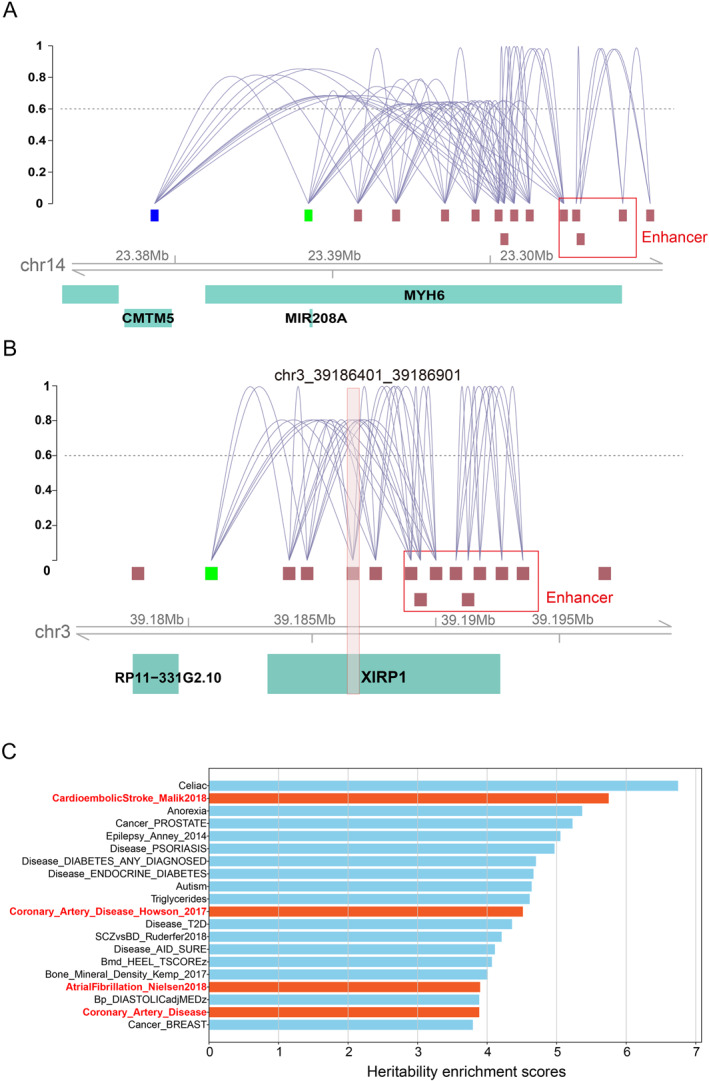
CASHeart facilitates the analysis of cardiac‐specific gene regulation and genetic variation. (A) Co‐accessibility relationships of peaks surrounding the MYH6 gene, where connections with co‐accessibility less than 0.6 have been filtered out. The red box highlights the enhancer region provided by GeneCards, while the two peaks marked in blue and green fall within two annotated regions. (B) Co‐accessibility relationships around a differentially accessible peak in cardiomyocytes, with connections having co‐accessibility less than 0.6 filtered out. This peak falls within the X1RP1 gene region and the red box represents the enhancer region provided by GeneCards. (C) The top 20 results from the genetic enrichment scores calculated for 130 diseases or traits using the LDSC method, of which 4 items are related to the heart.

As an example, we examined the MYH6 gene, which is specifically expressed in cardiomyocytes. This gene encodes the α‐heavy chain subunit of cardiac myosin and is one of the main components of cardiac myofibrils. We queried GeneCards [30] to locate a known enhancer for this gene within the region marked by the red box in Figure 5A. The peak in this region exhibited strong co‐accessibility with several upstream peaks. It is worth noting that the peak marked in green is located within MIR208A, which corresponds to a segment of noncoding miRNA that participates in post‐transcriptional regulation of gene expression by influencing mRNA stability and translation, thereby revealing regulatory relationships within the MYH6 gene. The peak marked in blue is located within the CMTM5 gene, which encodes a multi‐channel membrane protein that is functionally related to the protein encoded by the MYH6 gene. In addition, we analyzed the regulatory significance of the peaks identified through the analysis of differentially accessible chromatin regions. Taking the region of chr3_39186401_39186901 as an example, as shown in Figure 5B, this region is located within the XIRP1 gene, the protein encoded by this gene is a striated muscle protein and belongs to the Xin actin‐binding repeat‐containing protein family. The co‐accessibility information indicates that the enhancer region provided by GeneCards exhibits co‐accessibility with the peaks at the promoter region of this gene, highlighting the regulatory role of enhancers on promoters [31].

To validate the capacity of CASHeart in the study of genetic variants related to complex phenotypes based on the cell‐type‐specific peaks, we implemented the heritability enrichment analysis using partitioned linkage disequilibrium score regression (LDSC) [32]. In detail, we adopted LDSC to estimate the heritability of 130 complex traits and diseases by examining the enrichment of genetic variants within the peaks provided by CASHeart. Among the 130 traits and diseases included in the analysis, 5 are closely related to the heart. We focused on examining how many of these heart‐related traits and diseases ranked in the top 20 based on their genetic enrichment scores. As shown in Figure 4, 5 out of the top 20 ranked traits and diseases are associated with the heart, a result that is statistically significant by one‐sided binomial exact test (*p*‐value = 0.00532). This indicates that the peaks provided in CASHeart have a high degree of overlap with genetic variation sites associated with heart diseases. In addition, we performed SNPsea analysis [33] to identify the tissues affected by the identified risk loci within the peaks provided by CASHeart. As shown in Figure S2, in addition to common immune cells, cardiac myocytes and smooth muscle cells showed significant results in the analysis. Altogether, the peaks provided by CASHeart are enriched with variants related to the heart and cardiac disease, illustrating the tissue specificity and functional importance of these peaks.

## 3 CONCLUSIONS AND DISCUSSION

In this study, we introduced CASHeart, a comprehensive database of human heart single‐cell chromatin accessibility data, designed to address the limitations of current datasets in terms of scale, quality, and integration. The primary goal of CASHeart is to provide a standardized and unified resource that can enhance the analysis and understanding of the epigenomic landscape of the human heart. Our results demonstrate that CASHeart not only offers a more refined view of heart cell type heterogeneity but also improves the accuracy of differential chromatin accessibility analyses and differential gene expression analyses compared to the original datasets.

One of the key findings is that by incorporating uniformly processed data of CASHeart as a reference, the dimensionality reduction and clustering outcomes for external datasets were significantly enhanced. This indicates the potential of CASHeart to serve as a valuable reference for improving the quality of analyses in studies regarding single‐cell chromatin accessibility and transcriptomic data. In addition, the integration of transformed gene activity scores further facilitates cross‐modal analysis, providing a more holistic understanding of gene regulation in heart tissues. The development of the CASHeart online platform ensures that this rich dataset is accessible and useable for the broader research community. With features such as browsing, searching, and downloading of data, the platform is positioned to be a critical resource for advancing research in heart development, disease mechanisms, and therapeutic responses.

Although there are already some similar or related databases, such as Heart Cell Atlas v2 [9], CHDgene [34], ATACdb [35], and Cistrome Data Browser [36], they mainly focus on transcriptomic data, specific disease‐related genes, or overall chromatin accessibility regions. They lack in‐depth analysis and detailed annotation of single‐cell chromatin accessibility data specific to the human heart. CASHeart fills this gap by focusing on the integration and analysis of single‐cell chromatin accessibility data in the human heart, offering comprehensive functional annotations and data distribution information. It serves as a robust resource platform for studying chromatin regulatory mechanisms in the heart. Despite its advancements, CASHeart has several limitations. The current database scale is limited and will benefit from further expansion to include additional datasets. Batch effects arising from variations in experimental conditions and sequencing methods across different studies present significant challenges, and while efforts to correct these effects have been made, complete normalization remains difficult. Additionally, some data could not be integrated due to missing sequencing fragments. Looking ahead, we aim to address these limitations by continually updating and expanding the database with new data, enhancing its coverage and accuracy.

In summary, CASHeart represents a significant advancement in the availability and utility of single‐cell chromatin accessibility data for the human heart. By providing a large‐scale, high‐quality, and integrated dataset, CASHeart is expected to greatly enhance future research efforts in cardiovascular epigenomics, offering new insights into the molecular mechanisms underlying heart health and disease.

## 4 MATERIALS AND METHODS

### 4.1 Data collection

The HGCA dataset is derived from a single‐cell chromatin accessibility atlas of adult whole‐body tissues, with sequencing data available from the Gene Expression Omnibus under accession number GSE184462. The FCA dataset is sourced from a single‐cell chromatin accessibility atlas of infant whole‐body tissues, with sequencing data stored under accession number GSE149683. The Kanemaru2023 dataset is available from the online data portal provided in the original publication, accessible at Heartcellatlas V2 website. The processed Hocker2021 dataset is from GSE165837. The Wang2020 dataset is available under GSE109816 and the Tucker2020 dataset is accessible via the Broad Institute’s Single Cell Portal, with study accession number SCP498.

### 4.2 Data processing

For the sequencing fragment files collected from all samples, we first standardized the genome version by converting the genome version of the five FCA samples from GRCh37 (hg19) to GRCh38 (h38). Subsequently, barcodes lacking cell type annotations were filtered out. We then performed peak calling and quality control using snapATAC2 version 2.6.4. Specifically, we used the built‐in MACS3 algorithm in snapATAC2 for peak calling, followed by calculating the Tn5 integration‐based sequence specificity enrichment (TSSE) score for each cell. Cells with a TSSE score greater than or equal to 7 were retained, and doublets were further filtered out. To address potential barcode duplication across different samples, sample name prefixes were added for differentiation. Cell annotations were primarily derived from metadata files provided in the original publications, annotated based on sample names and barcodes. The final single‐cell chromatin accessibility data were stored as AnnData objects [37]. The single‐cell gene activity scores were processed using EpiScanpy version 0.3.2 through the *GeneActivity* function. In addition to the necessary single‐cell chromatin accessibility data, genome annotation files from NCBI in hg38 version were provided, with the upstream length of gene promoter regions specified as 5000 bp.

### 4.3 Differential chromatin accessibility and differential gene expression analysis

First, cell types were standardized manually, including unifying annotations for case sensitivity and different expressions for the same type. To ensure more accurate calculation of differential open regions and differential gene expression for each cell type, we filtered out types with fewer than 100 cells. Then, chromatin accessibility data were processed using TF‐IDF and PCA, while gene expression data were processed with read normalization and log transformation. Subsequently, *t*‐tests were used to calculate the differential features between each type of data and the data from other types, and *p*‐values were used to identify differential open regions and differential expressed genes for each cell type. Specifically, we employed the *rank_genes_groups* and *rank_feature_groups* methods provided by Scanpy [38] and EpiScanpy for implementation.

### 4.4 Using CASHeart as a reference dataset

For chromatin accessibility data, the first step is to map the target dataset to the peaks provided by CASHeart. Specifically, we use BEDTools [39] to compute the intersection between the peaks in the CASHeart data and those in the target dataset. For each peak in CASHeart, we replace it with the first overlapping peak from the target dataset. After standardizing the peaks, we concatenate the reference dataset with the target dataset and perform TF‐IDF and PCA processing on the combined dataset. Subsequently, we extract the cells from the target dataset for the clustering and calculation of clustering metrics. For gene activity scores, it is necessary to standardize the genes between the reference dataset and the target dataset. During the selection of highly variable genes, we also retain the highly variable genes from the original target dataset. These datasets are then subjected to normalization and other preprocessing steps, followed by similar extraction and clustering of cells from the target dataset.

### 4.5 Calculation of clustering metrics

In this study, three clustering metrics were primarily used: AMI, ARI, and Homogeneity (Homo) [40]. AMI adjusts the mutual information to account for the possibility of random clustering, providing a normalized measure of similarity. The range of AMI is [0, 1], where one indicates identical clustering and 0 indicates completely independent clustering. ARI is an adjustment of the traditional Rand Index that eliminates the effects of random clustering, allowing its values between 0 and 1 to better reflect the actual effectiveness of clustering. The Homogeneity metric evaluates the internal consistency of each cluster in a clustering result, specifically whether the samples within the same cluster all belong to the same true class. Before calculating these metrics, clustering is first performed on the processed single‐cell chromatin accessibility data using the Leiden and Louvain methods to obtain clustering labels. Then, functions such as *episcanpy.tl.ARI* integrated in EpiScanpy are used to directly compute the metrics.

### 4.6 Analysis of gene regulation and genetic variations

We extracted data from CASHeart based on cell types and used EpiScanpy to select the top 100,000 peaks. Following the Cicero tutorial, we calculated co‐accessibility between different peaks using default parameters and visualized the results with the provided plotting tools [41]. We conducted SNP enrichment analysis using SNPsea with default settings, which allowed us to identify tissues affected by the peaks from CASHeart. The enrichments of tissue‐specific expression profiles for 17,581 genes across 79 human tissues [42] were quantified for the peaks, and the top 30 significantly enriched tissues were displayed. Additionally, we performed heritability enrichment analysis using LDSC with default settings to quantify the enrichment of heritability for heart‐related phenotypes within the peaks from CASHeart. Using HapMap3 SNPs and GWAS summary statistics obtained from the Broad LD Hub, LDSC estimated heritability enrichment using European samples from the 1000 Genomes Project as the linkage disequilibrium reference panel [43].

## AUTHOR CONTRIBUTIONS

**Qun Jiang**: Methodology; Resources; Validation; Visualization; Writing ‐ original draft. **Xiaoyang Chen**: Data curation; Formal analysis; Methodology; Resources; Writing ‐ original draft. **Zijing Gao**: Data curation; Formal analysis; Methodology; Resources; Software; Writing ‐ review & editing. **Jinmeng Jia**: Conceptualization; Formal analysis; Investigation; Resources; Validation; Writing ‐ review & editing. **Shengquan Chen**: Conceptualization; Investigation; Project administration; Software; Supervision; Writing ‐ review & editing. **Rui Jiang**: Conceptualization; Funding acquisition; Project administration; Supervision; Writing ‐ review & editing.

## CONFLICT OF INTEREST STATEMENT

The authors declare no conflicts of interest.

## ETHNICS STATEMENT

This article does not contain any studies with human or animal materials performed by any of the authors.

## Supporting information

Supporting Information S1

## Data Availability

All data mentioned in this article are referenced in Section 4.1 and are publicly available on the internet.
