# Supplementary material for: CASHeart: A database of single cells chromatin accessibility for the human heart
Source: Quant Biol. 2025 Feb 12;13(2):e90. doi: 10.1002/qub2.90 (PMC12806100; doi:10.1002/qub2.90)
Supplement: Supplementary file 1 — Supporting Information S1 [file QUB2-13-e90-s001.docx]

**CASHeart: a database of single cells chromatin accessibility for the human heart**

Qun Jiang^1^, Xiaoyang Chen^1^, Zijing Gao^1^,
Jinmeng Jia^1^, Shengquan Chen^2^, Rui Jiang^1,*^

^1^ Ministry of Education Key Laboratory of Bioinformatics, Bioinformatics Division at the Beijing National Research Center for Information Science and Technology, Center for Synthetic and Systems Biology, Department of Automation, Tsinghua University, Beijing 100084, China

^2^ School of Mathematical Sciences and LPMC, Nankai University, Tianjin 300071, China

* To whom correspondence should be addressed.

Corresponding author contact information: ruijiang@tsinghua.edu.cn


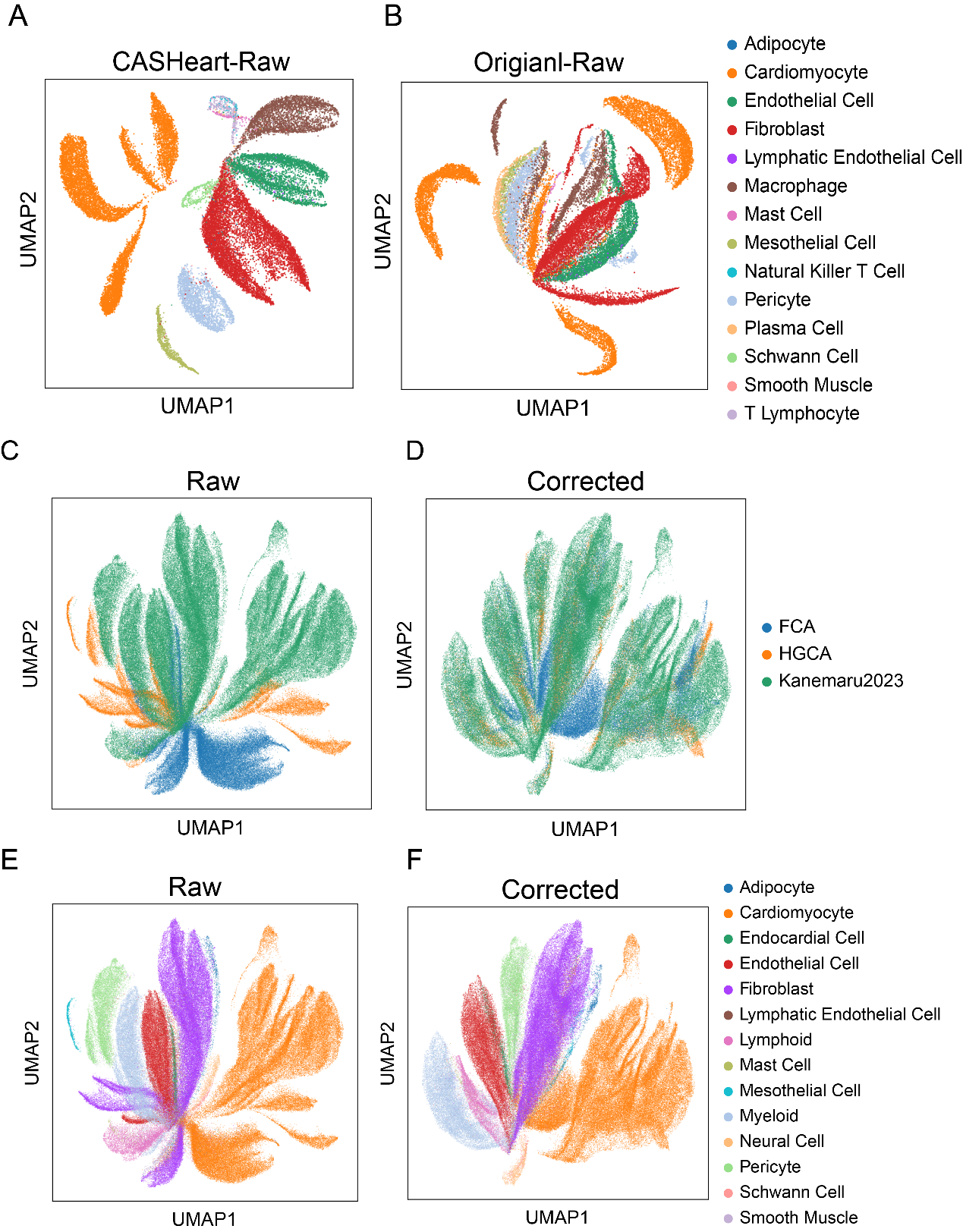


**Supplementary Figure 1.** A. UMAP visualization of the HGCA dataset processed by CASHeart before batch correction, with colors representing cell types. B. UMAP visualization of the original HGCA dataset before batch correction, with colors representing cell types. C. UMAP visualization of all CASHeart data colored by dataset labels without batch correction. D. UMAP visualization of all CASHeart data colored by cell type labels without batch correction; E. UMAP visualization of all CASHeart data colored by dataset labels with Harmony batch correction; F. UMAP visualization of all CASHeart data colored by cell type labels with Harmony batch correction.


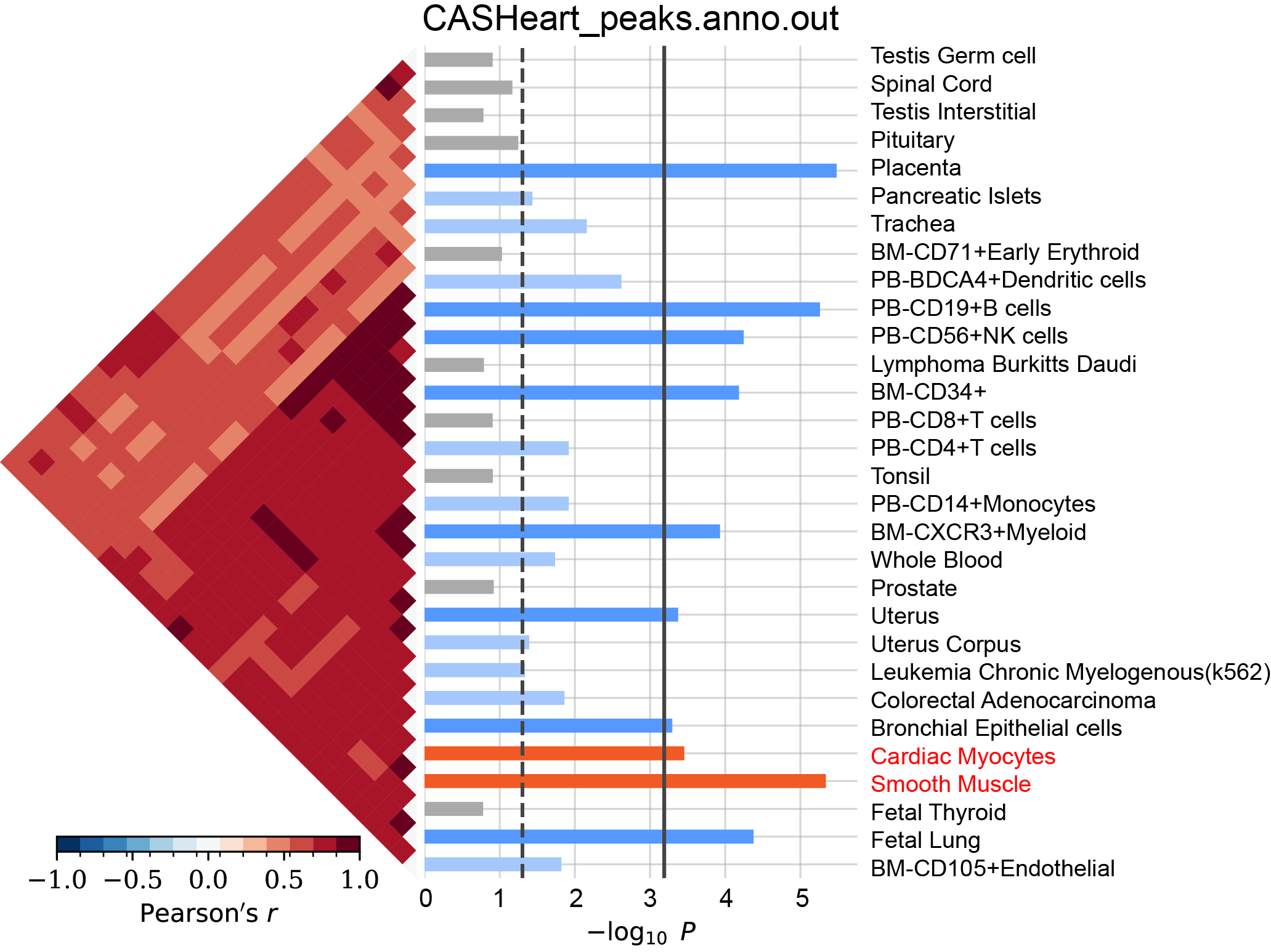


**Supplementary Figure 2.** Top 30 most significantly enriched tissues identified by SNPsea on peaks of CASHeart. The vertical dashed lines represent the one-sided P-value cutoffs at the 0.05 level, whereas the solid lines denote the cutoffs at the 0.05 level for the one-sided P-value with Bonferroni correction. The expression profiles were ordered using hierarchical clustering with the unweighted pair-group method with arithmetic means.
